# Comparative Evaluation of Two Bioceramic-Based Sealers on Root Dentin Strength and Fracture Resistance Under Varying Tooth Conditions: An In Vitro Study

**DOI:** 10.7759/cureus.117603

**Published:** 2026-10-07

**Authors:** Heba Allah Ateyah, Helen R Ayoubi, Yasser Alsayed Tolibah

**Affiliations:** 1 Department of Endodontics and Operative Dentistry, Damascus University, Damascus, SYR; 2 Department of Pediatric Dentistry, Damascus University, Damascus, SYR

**Keywords:** bc hiflow sealer, bioceramic sealer, fracture resistance, reinforce root dentin, vertical root fracture

## Abstract

Objectives

This study aimed to compare the effects of TotalFill Bioceramic (BC) Sealer (FKG Dentaire, La Chaux-de-Fonds, Switzerland) and TotalFill BC HiFlow Sealer (FKG Dentaire, La Chaux-de-Fonds, Switzerland) on the fracture resistance of roots with and without incomplete simulated vertical root fractures (VRFs).

Methods

Ninety single-rooted, single-canaled human permanent premolars were standardized to a root length of 15 mm and randomly allocated into three main groups (n=90 in total) and six subgroups (n=15 each) according to the treatment and the presence or absence of a simulated incomplete VRF. Group A (control) received neither canal preparation nor obturation and comprised A1, roots without VRF (negative control), and A2, roots with VRF (positive control). Group B was prepared and obturated with TotalFill BC Sealer using the single-cone technique and comprised B1 (without VRF) and B2 (with VRF). Group C was prepared and obturated with TotalFill BC HiFlow Sealer using warm vertical compaction and comprised C1 (without VRF) and C2 (with VRF). Incomplete VRFs, 7.5 mm long and 0.25 mm wide, were created on the buccal surface starting from the apex. All specimens were loaded vertically in a universal testing machine with a 1.5 mm cylindrical steel rod at 1 mm/min until fracture, and the peak force was recorded in Newtons (N). Data were analyzed using two-way analysis of variance (ANOVA) with treatment and VRF status as independent factors (α=0.05).

Results

The mean fracture resistance was highest in the negative control group (A1: 853.83±173.63 N), followed by the positive control group (A2: 827.30±167.73 N), the TotalFill BC groups without and with VRF (B1: 726.35±226.00 N; B2: 683.70±198.18 N), and the TotalFill BC HiFlow groups without and with VRF (C1: 538.92±198.32 N; C2: 531.78±84.48 N). Two-way ANOVA revealed no significant main effect of treatment (F(2,54)=2.54; p=0.088) or of incomplete VRF (F(1,54)=0.98; p=0.327) and no significant interaction between them (F(2,54)=0.60; p=0.553).

Conclusion

Within the limitations of this in vitro study, neither TotalFill BC Sealer nor TotalFill BC HiFlow Sealer significantly altered root fracture resistance compared with untreated roots, and the presence of a simulated incomplete VRF had no significant effect. Although mean values were lower in the HiFlow groups, these differences were not statistically significant. Larger studies are needed to confirm these findings.

## Introduction

The primary objective of endodontic therapy is to preserve natural teeth within the dental arch and prevent their extraction, thus maintaining optimal oral function and esthetics [1]. Reinforcing dental tissues is also critical to improve fracture resistance and ensure long-term tooth integrity [2]. However, various reasons may ultimately necessitate tooth extraction, with dental fractures being among the most critical causes, whether coronal or radicular. Root fractures are categorized as vertical or transverse [3]. Vertical root fractures (VRFs) are longitudinal cracks that develop along the surfaces of dental roots [4], accounting for approximately 2-5% of all crown-root fractures [5]. These fractures can affect one surface of the root (known as incomplete vertical fractures) or involve both surfaces (referred to as complete vertical fractures). Maxillary and mandibular premolars, as well as the mesial roots of mandibular molars, are especially susceptible [4]. Although VRFs most frequently occur in endodontically treated teeth, they can also occur in vital teeth [6]. The causes of VRFs in endodontically treated teeth are highly complex and multifactorial. No single definitive cause exists, making VRF prevention extremely difficult. A variety of factors contribute to this issue, encompassing both uncontrollable predisposing factors and direct causative factors [7].

Bioceramic-based root canal sealers have garnered considerable attention in endodontics due to their advantageous biological and mechanical properties. These materials are highly biocompatible and have intrinsic antibacterial activity, which helps to prevent microbial colonization within the root canal system [8]. Furthermore, their bioactive components release calcium and hydroxyl ions and can promote hydroxyapatite formation at the sealer-dentin interface, contributing to their biological performance and sealing ability [8]. From a mechanical standpoint, some in vitro studies have reported increased fracture resistance of roots filled with calcium silicate-based sealers [9,10], which has been attributed to their slight setting expansion and their ability to form a mineral infiltration zone within dentin. However, this evidence remains inconsistent, and bioactivity itself should not be equated with mechanical reinforcement. 

In roots with an incomplete VRF, the root canal sealer may play a role beyond conventional obturation. The fracture line provides a pathway between the root canal system and the periodontium through which bacteria and their by-products can spread, leading to periodontal breakdown and, ultimately, tooth loss [11]. A sealer that flows into the fracture line and seals it may therefore limit this bacterial communication. From a mechanical perspective, an incomplete fracture acts as a stress concentrator, with high stresses developing at the crack tip under occlusal loading, which may promote further crack propagation [10]. A sealer that fills the fracture space and adheres to its walls could theoretically distribute occlusal stresses more evenly, reduce stress concentration at the crack tip, and thereby slow fracture propagation [10]. Calcium silicate-based sealers are of particular interest in this regard because of their flowability, slight setting expansion, and ability to bond to dentin through the formation of a mineral infiltration zone [10,11]. However, whether these properties translate into improved fracture resistance of roots with incomplete VRFs has been investigated in only a few in vitro studies [10].

Although bioceramic sealers have been shown to reinforce intact endodontically treated roots, their ability to reinforce roots already affected by incomplete VRFs remains largely unexplored. Moreover, direct comparisons between TotalFill Bioceramic (BC) Sealer and its high-flow counterpart (BC HiFlow) (FKG Dentaire, La Chaux-de-Fonds, Switzerland) under these conditions are scarce, and to the authors' knowledge, limited evidence is available evaluating these conditions within a single experimental design. Addressing this gap is clinically relevant, as incomplete VRFs represent a borderline condition where improvement in mechanical fracture resistance may be of potential clinical interest.

In this context, this study aimed to evaluate the impact of two bioceramic-based root canal sealers, namely, TotalFill BC Sealer and TotalFill BC HiFlow Sealer, on the fracture resistance of roots with simulated incomplete VRFs. The effects were further compared with those observed in non-fractured endodontically treated roots filled with the same sealers. Additionally, outcomes were assessed relative to natural, untreated teeth, encompassing both non-fractured teeth and teeth with incomplete VRFs. This comprehensive approach provides a detailed evaluation of bioceramic-based sealers' potential to reinforce root dentin and improve fracture resistance across a range of conditions, from compromised to healthy natural teeth.

## Materials and methods

Study samples

This in vitro study was conducted at the Department of Endodontics and Operative Dentistry at the Faculty of Dentistry, Damascus University, in Damascus, Syria, from December 2023 to December 2024. The institution's Local Research Ethics Committee approved the research (approval number: 382; approval date: 15-03-2022). The sample consisted of 90 freshly extracted human permanent premolars collected from the Department of Oral Surgery at Damascus University and removed due to caries or periodontal disease. Soft tissue residue was removed from the extracted teeth, which were then stored in saline at 4°C for use within one month of extraction. Patients provided informed consent to include their extracted teeth in the study.

The sample size was calculated using G*Power (Version 3.1.9.4, Heinrich Heine University Düsseldorf, Düsseldorf, Germany). For the one-way analysis of variance (ANOVA) test, the following parameters were applied: 95% study power, an alpha level (α) of 0.05, and an effect size of 0.53. These inputs were derived from a study by Demiriz and Bodrumlu [10] that used a similar methodology.

Tooth preparation

Only premolars with a single canal classified as type 1 according to the Vertucci classification [12] were included in this study. Teeth exhibiting an open apex, resorption, curved canals, or previous endodontic treatment were excluded. Mesiodistal digital radiographs were obtained for all specimens to assess root canal anatomy and to exclude teeth with dentin thickness of less than 1 mm. Subsequently, all teeth were examined under a stereomicroscope (Model SKT 41321; Meiji Techno, Saitama, Japan) at 20× magnification to exclude specimens with cracks or fractures.

The teeth were standardized to a root length of 15 mm by removing excess coronal and apical portions using a diamond disc with water cooling. The specimens were randomly assigned to three main groups (n=30) based on filling material, and each main group was randomly divided into two subgroups (n=15) according to the presence or absence of a VRF. Group 1 (A) served as the control group, in which the root canals were neither instrumented nor obturated, and was divided into A1 without VRF (negative control) and A2 with VRF (positive control). Group 2 (B), the first experimental group, included teeth obturated with TotalFill BC Sealer and gutta-percha and was subdivided into B1 without VRF and B2 with VRF. Group 3 (C), the second experimental group, included teeth obturated with TotalFill BC HiFlow Sealer and gutta-percha and was subdivided into C1 without VRF and C2 with VRF.

Thirty root specimens were randomly selected and assigned to the control groups; no root canal preparation was performed for these samples. The remaining 60 specimens underwent root canal preparation using the AF Gold system up to a master apical file size 25 with a 0.04 taper (AF Gold, Fanta, Shanghai, China). The root canal was irrigated using 5 mL of 5.25% sodium hypochlorite (Ibn Al-Haytham, Damascus, Syria) after each file exchange.

Simulating incomplete VRFs

After the completion of root canal preparation, artificial incomplete VRFs were induced in half of the specimens. This simulation technique was developed by the first author (HAA) following a pilot study aimed at achieving the most accurate simulation of incomplete VRFs.

The induced defects originated at the apex and extended coronally for approximately half the root length (7.5 mm), with a standardized width of 0.25 mm. These standardized defects, which served as a simulated model of an incomplete VRF, were confined to the buccal surface and extended toward the root canal space without involving the lingual wall.

To ensure consistency across specimens, the internal and external fracture line lengths were standardized. A reference line was drawn on the buccal surface of each root using a thin permanent marker pen, extending 7.5 mm coronally from the apex. The length was then verified with a digital caliper (PowerAxis, USA, manufactured in China). A single polymethyl methacrylate (PMMA) fiber-optic strand was inserted into the root canal and retained throughout the procedure to guide fracture formation (Figure 1).

**Figure 1:**
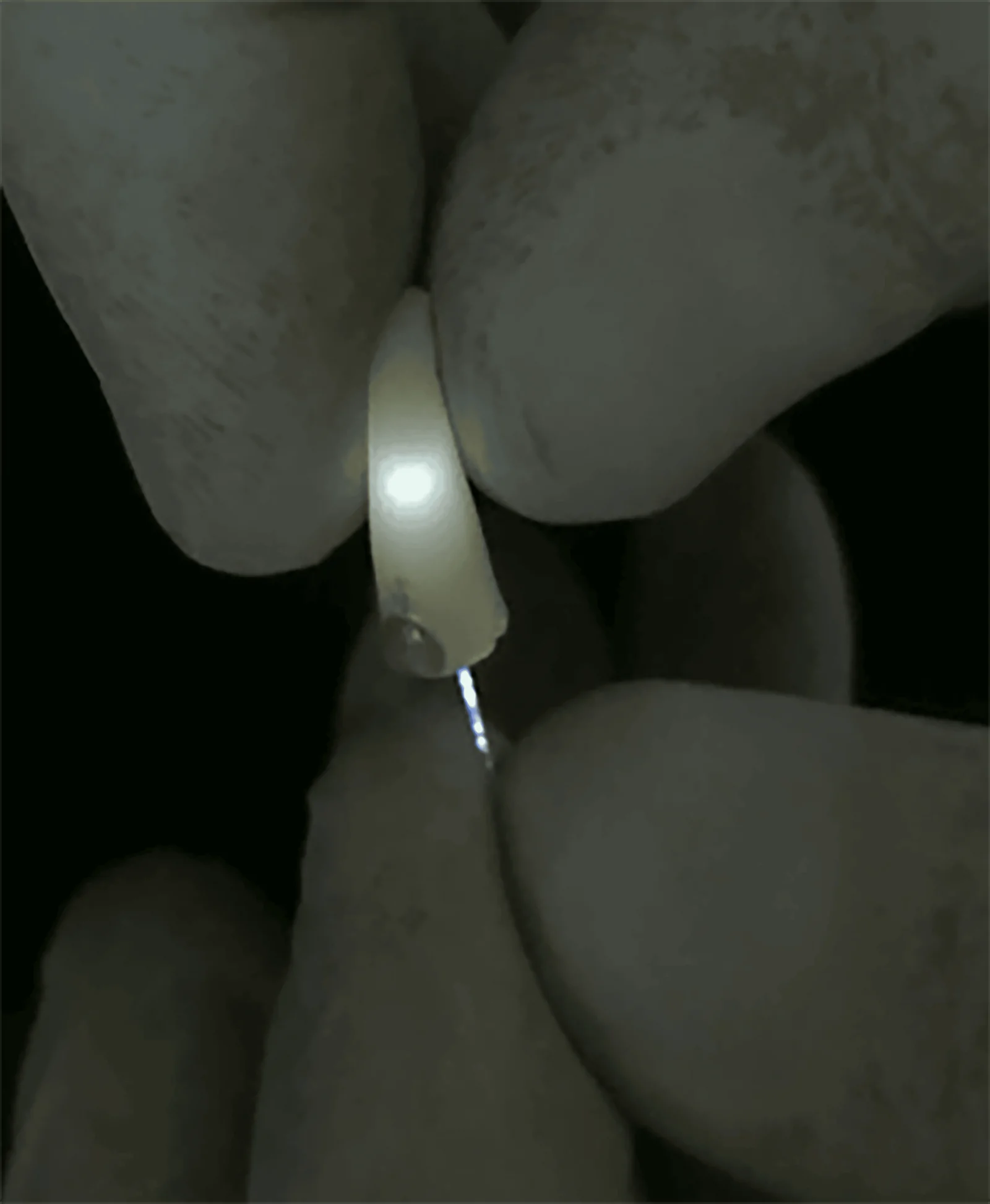
Inserting a single polymethyl methacrylate (PMMA) plastic fiber-optic strand into the root canal during the process

Initial grooves were created using a 0.25 mm diamond disc (Hager & Meisinger GmbH, Neuss, Germany) under copious water cooling, progressing coronally from the apex along the marked line. A modified G2 supragingival ultrasonic scaler (Woodpecker Medical Instrument, Guilin, China) was then used to complete the fracture by removing the remaining dentin that the diamond disc could not access (Figure 2).

**Figure 2:**
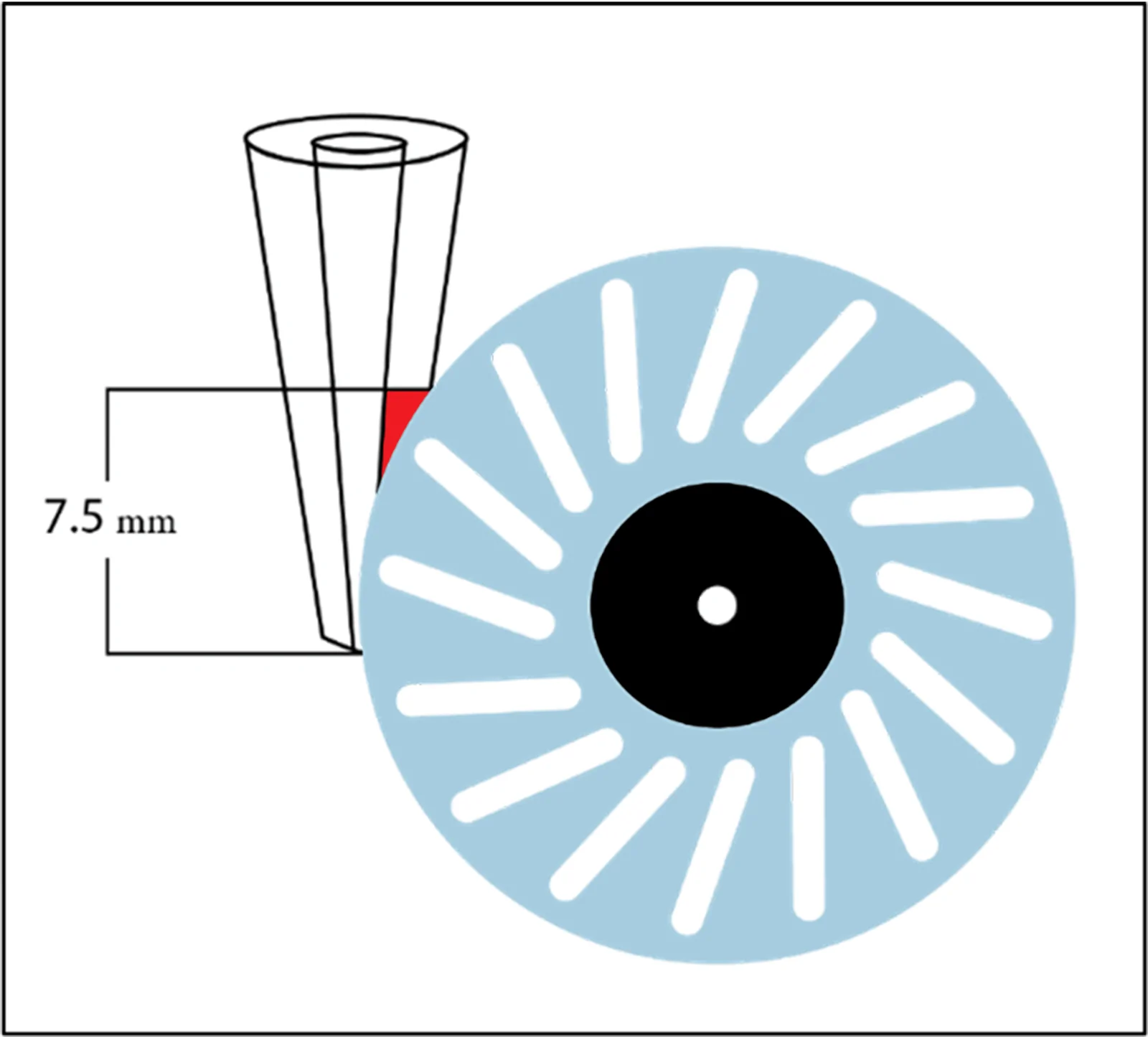
Schematic representation of the limitation of the diamond disc in accessing the entire dentin thickness during the simulation of an incomplete vertical root fracture. The red-colored area represents the remaining dentin tissue that could not be reached by the diamond disc and therefore required completion using the modified ultrasonic tip. The simulated fracture extended approximately 7.5 mm coronally from the root apex The figure was created using Adobe Illustrator Version 25.0 (64-bit) (Adobe Inc., San Jose, California, United States).

The tip was modified to reduce its thickness to 0.25 mm over its apical 4 mm. It was mounted on a piezoelectric device set at power level 9. The tip was carefully advanced coronally along the marked line, perpendicular to the buccal surface toward the canal space, until the fiber-optic light became visible along the entire fracture line, confirming that both internal and external fracture lines were of equal length. Finally, all specimens were examined under a stereomicroscope to confirm the absence of additional cracks.

After the simulation of the incomplete VRF, the smear layer was removed by irrigating the samples with 5 mL of 17% ethylenediaminetetraacetic acid (EDTA) solution (Aphamea, Hama, Syria) for one minute. Final irrigation was performed with 5 mL of 5.25% NaOCl and 5 mL of sterile saline.

Subsequently, the appropriate master gutta-percha cones were selected for each canal by confirming that the cone reached the full working length with slight resistance upon withdrawal (tug-back). After irrigation, all treated roots were dried using sterile paper points (Orodeka, Shandong, China).

Obturation of the experimental groups

In Groups B1+B2 and C1+C2, a total of 60 root samples were obturated using a gutta-percha cone (Orodeka, Shandong, China) in combination with TotalFill BC-based Sealers, in accordance with the manufacturer's instructions. In Groups B1+B2, TotalFill BC Sealer was applied using the single-cone obturation technique, whereas in Groups C1+C2, TotalFill BC HiFlow Sealer was employed with the warm vertical compaction technique. After obturation, all samples were evaluated using periapical mesiodistal radiographs to assess the quality of the root canal filling and the radiographic distribution of the sealer within the simulated fracture defects (Figures 3-4).

**Figure 3:**
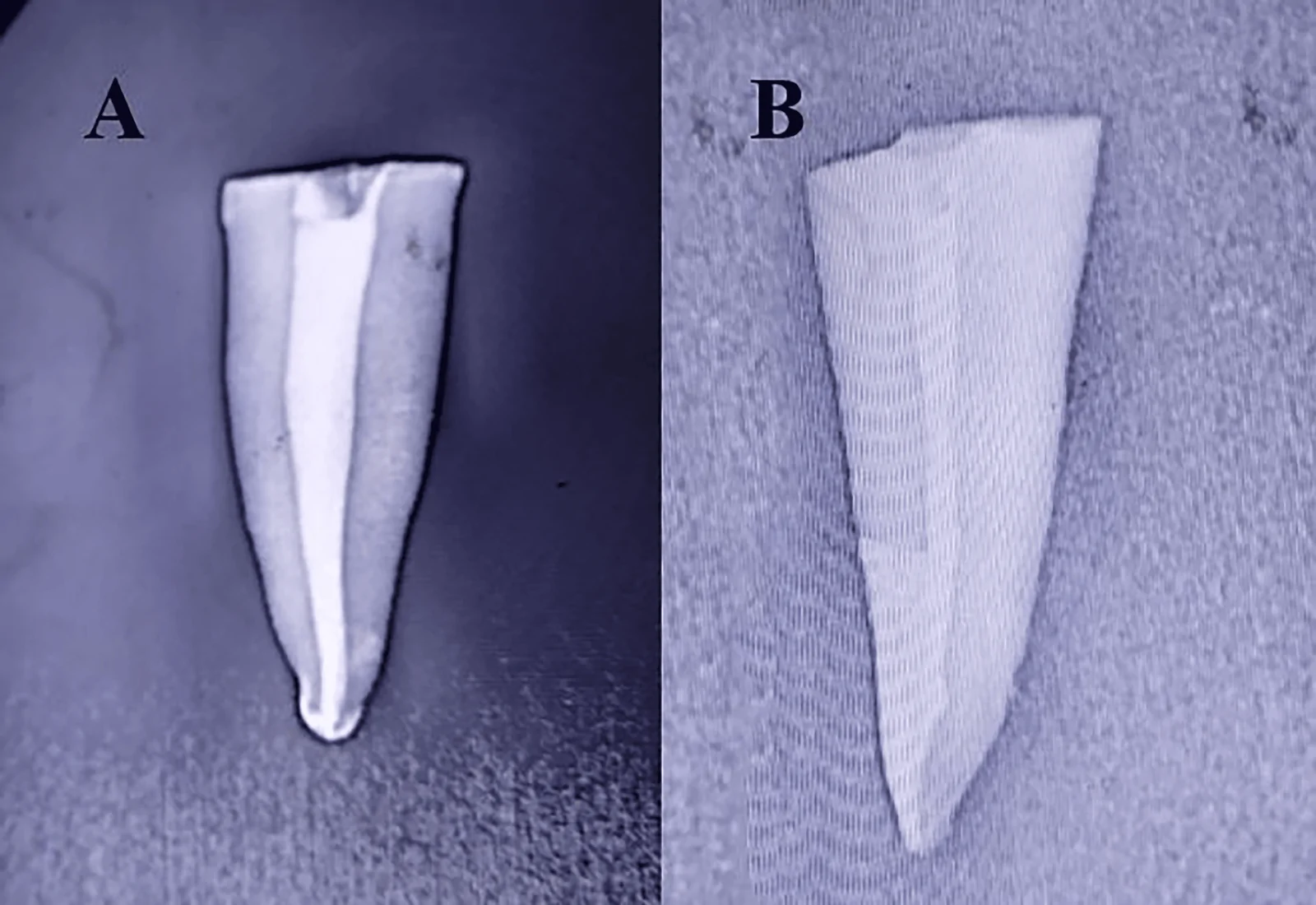
Postoperative radiograph showing the root canal filling and radiopaque sealer within the simulated fracture defect: (A) Group B1 and (B) Group B2

**Figure 4:**
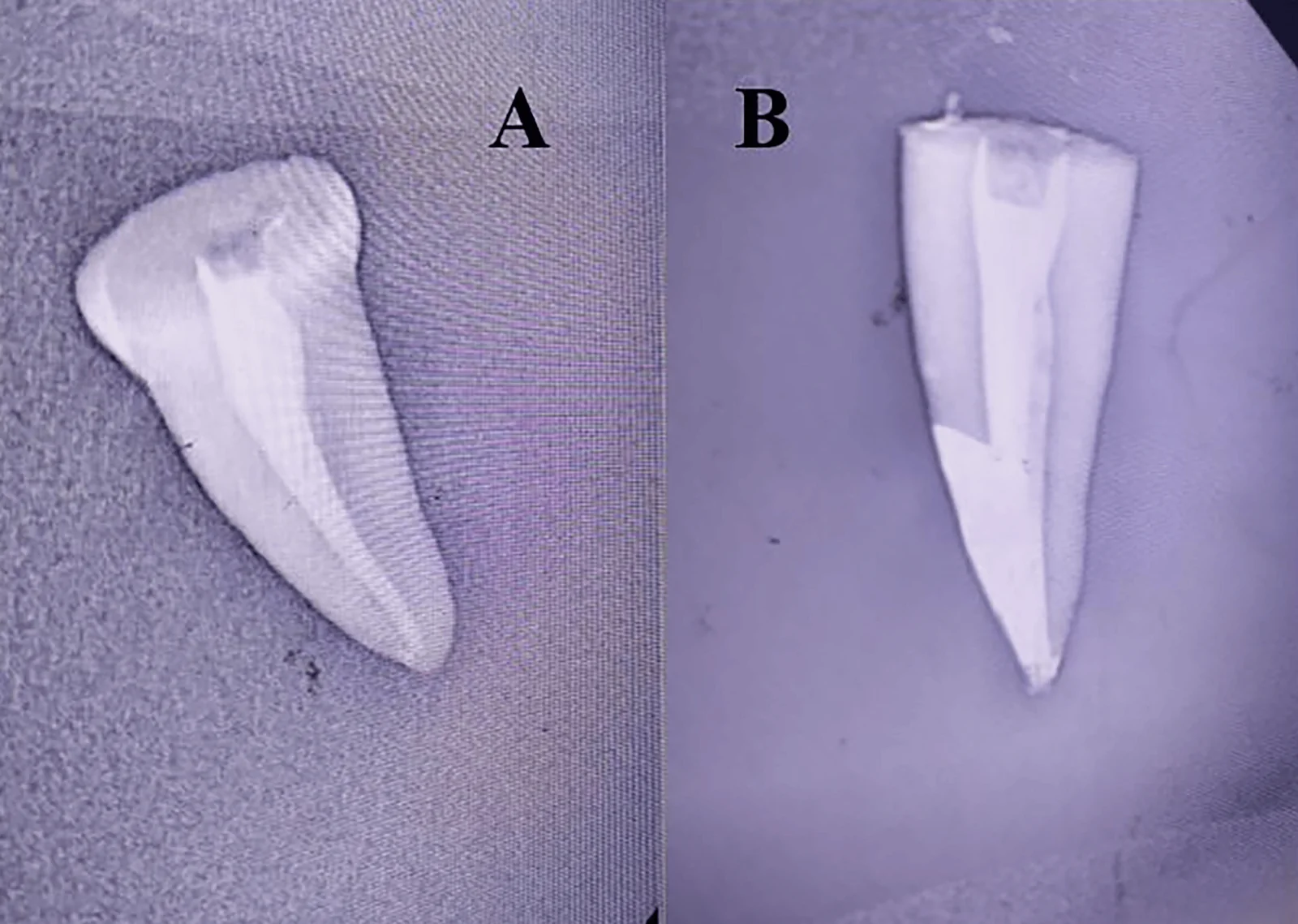
Postoperative radiograph showing the root canal filling and radiopaque sealer within the simulated fracture defect: (A) Group C1 and (B) Group C2

Excess root canal sealer and gutta-percha were meticulously removed from all teeth. Approximately 2 mm from the orifices was cleaned using a high-speed handpiece equipped with a diamond bur. The teeth were then restored coronally with light-cured glass ionomer cement (Fusion-I Seal, Prevest DenPro Limited, India), following the manufacturer's instructions. After restoration, all samples were stored in an incubator (Binder, Tuttlingen, Germany) at 37°C and 100% humidity for seven days to ensure complete setting of the sealers.

Next, the external surface of each root was coated with a liquid peel-off latex material (AL'VER, France), applied from the apex coronally while leaving the coronal 2 mm uncoated. This procedure simulated the periodontal ligament.

The coated portion of each root was positioned vertically and embedded in self-curing acrylic cubic blocks measuring 20×20×20 mm, with the coronal 2 mm of the uncoated root exposed above the block. Once the acrylic had fully set, the blocks were removed, and the samples were ready for fracture resistance testing (Figure 5).

**Figure 5:**
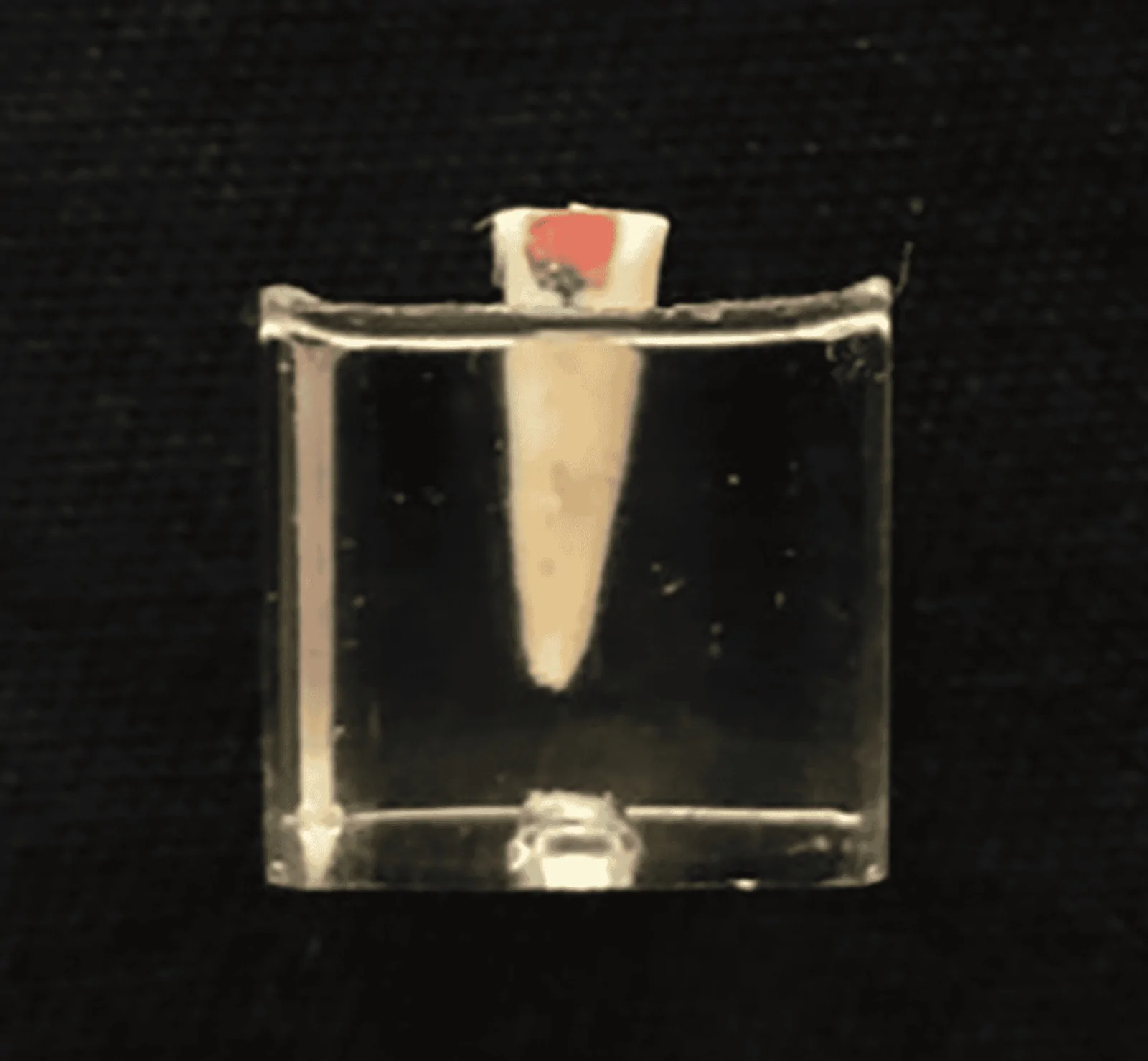
A sample in the acrylic cylinder blocks which was ready for the fracture test

Fracture test

The fracture resistance of each root was evaluated using an Instron Universal Testing Machine (Instron, Norwood, Massachusetts, United States). A cylindrical steel rod with a diameter of 1.5 mm was employed to apply a load directly to the center of the canal orifice, while the acrylic-embedded roots were positioned on the lower platform. Compressive force was applied along the long axis of the tooth at a constant rate of 1 mm/min until the root fractured. An independent examiner, who was unaware of the filling material used, documented the peak force that caused the fracture in Newtons (N).

Statistical analysis

Data were analyzed using IBM SPSS Statistics for Windows, Version 24.0 (IBM Corp., Armonk, New York, United States). Fracture resistance values (N) are presented as mean±standard deviation. The study followed a 3×2 factorial design, with treatment (no preparation or obturation, TotalFill BC Sealer, and TotalFill BC HiFlow Sealer) and simulated incomplete VRF (absent or present) as independent factors. The normality of data distribution within each subgroup was assessed using the Shapiro-Wilk test, and the homogeneity of variances was assessed using Levene's test. A two-way ANOVA was used to evaluate the main effects of treatment and simulated incomplete VRF, as well as their interaction, on fracture resistance, and effect sizes were reported as partial eta squared (η²p). Bonferroni-adjusted post hoc comparisons were planned in the event of a significant main effect or interaction. Because one subgroup showed a deviation from normality, the results were additionally verified using the Kruskal-Wallis test. The level of statistical significance was set at p<0.05.

## Results

Figure 6 illustrates the flowchart of the study.

**Figure 6:**
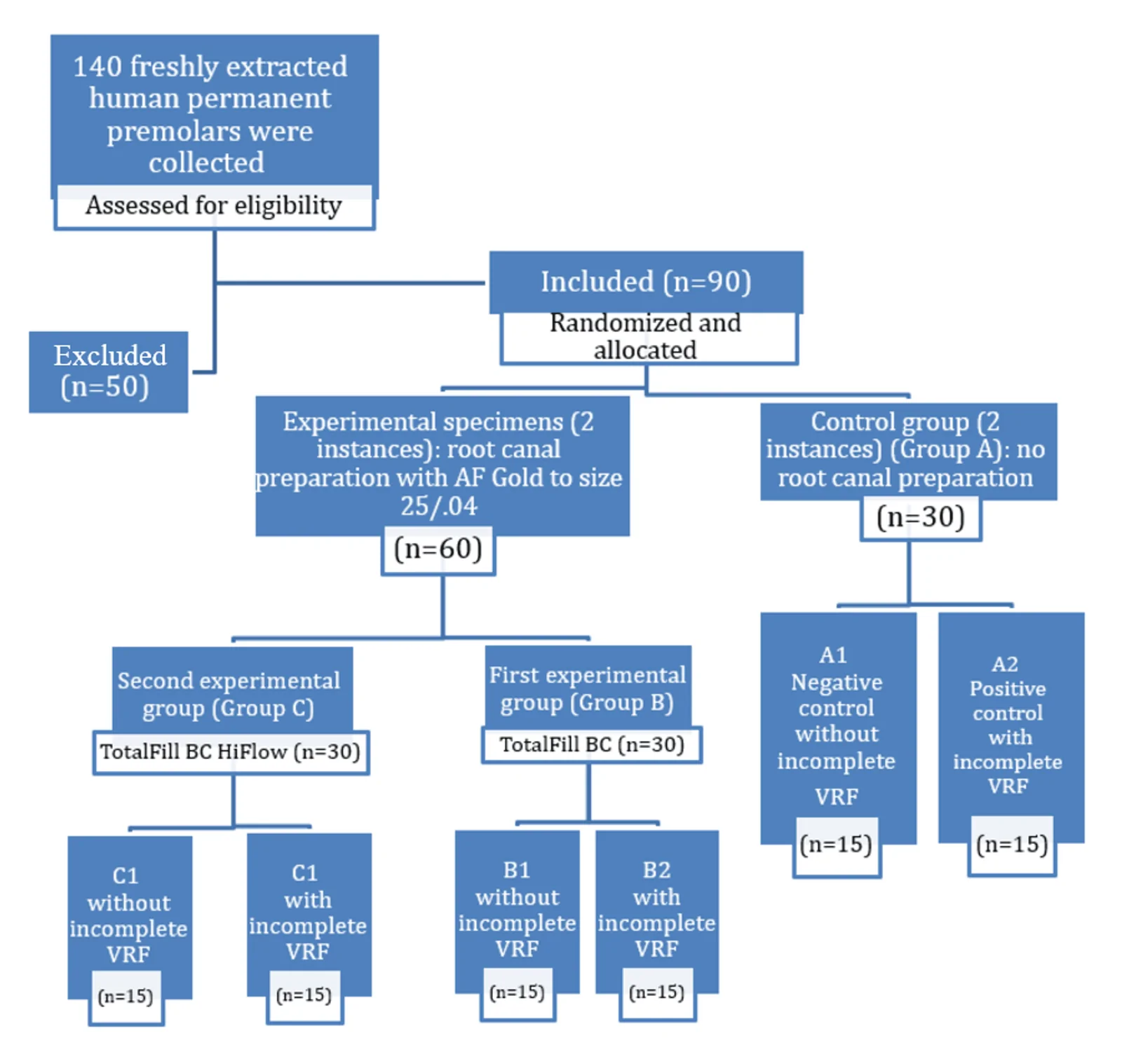
Study flowchart VRF: vertical root fracture

The mean fracture resistance was highest in the negative control Group A1, at 853.83±173.63. This was followed by the positive control Group A2 at 827.30±167.73, Group B1 at 726.35±226.00, Group B2 at 683.70±198.18, and Group C1 at 538.92±198.32. The lowest value was observed in Group C2 at 531.78±84.48 (Table 1).

**Table 1:** Descriptive statistics of fracture resistance values (N) in the studied subgroups VRF: vertical root fracture; SD: standard deviation; CI: confidence interval

Subgroup	n	Mean±SD (N)	95% CI (N)	Minimum (N)	Maximum (N)
A1: negative control (no VRF)	15	853.83±173.63	596.21-945.89	415.0	1203.0
A2: positive control (simulated VRF)	15	827.30±167.73	482.54-786.08	324.8	990.0
B1: TotalFill BC Sealer (no VRF)	15	726.35±226.00	564.68-888.02	308.0	990.0
B2: TotalFill BC Sealer (simulated VRF)	15	683.70±198.18	541.93-825.47	361.3	958.0
C1: TotalFill BC HiFlow Sealer (no VRF)	15	538.92±198.32	378.44-747.80	345.0	1180.0
C2: TotalFill BC HiFlow Sealer (simulated VRF)	15	531.78±84.48	466.55-684.61	368.8	938.0

The Shapiro-Wilk test indicated that the data were normally distributed in all subgroups except C1 (p=0.020), and Levene's test confirmed the homogeneity of variances (p=0.658). Two-way ANOVA revealed no statistically significant main effect of treatment (F(2,54)=2.54; p=0.088; η²p=0.086) or of simulated incomplete VRF (F(1,54)=0.98; p=0.327; η²p=0.018) and no significant interaction between the two factors (F(2,54)=0.60; p=0.553; η²p=0.022) (Table 2). Accordingly, post hoc comparisons were not performed. The absence of significant differences among the six subgroups was confirmed by the Kruskal-Wallis test (H(5)=7.65; p=0.177).

**Table 2:** Two-way ANOVA for the effects of treatment and simulated incomplete VRF on fracture resistance Significance level was set at p<0.05. df: degrees of freedom; η²p: partial eta squared; VRF: vertical root fracture; ANOVA: analysis of variance

Source of variation	Sum of squares	df	Mean square	F	P-value	η²p
Treatment	241,267.31	2	120,633.66	2.54	0.088	0.086
Simulated incomplete VRF	46,442.71	1	46,442.71	0.98	0.327	0.018
Treatment×simulated VRF	56,917.80	2	28,458.90	0.60	0.553	0.022
Error	2,564,792.63	54	47,496.16	-	-	-
Corrected total	2,909,420.45	59	-	-	-	-

When the data were pooled by factor, the mean fracture resistance was 702.68±233.53 N in the control group, 705.02±208.03 N in the TotalFill BC Sealer group, and 569.35±206.43 N in the TotalFill BC HiFlow Sealer group. Roots without a simulated incomplete VRF showed a mean fracture resistance of 686.84±251.67 N, compared with 631.20±188.11 N in roots with a simulated incomplete VRF (Table 3); none of these differences reached statistical significance.

**Table 3:** Mean fracture resistance values (N) according to each factor SD: standard deviation; VRF: vertical root fracture

Factor	Level	Mean±SD (N)
Treatment	A: Control (no preparation or obturation)	702.68±233.53
	B: TotalFill BC Sealer (single-cone)	705.02±208.03
	C: TotalFill BC HiFlow Sealer (warm vertical compaction)	569.35±206.43
Simulated incomplete VRF	Absent (A1, B1, C1)	686.84±251.67
	Present (A2, B2, C2)	631.20±188.11

## Discussion

The prognosis of teeth affected by VRFs is generally poor, and extraction is often the standard treatment. However, numerous case reports have described different approaches to preserve teeth with VRFs, with varying degrees of success [11]. These approaches emphasize cleaning the root canal system, three-dimensional obturation, sealing the fracture margins, and limiting further propagation of the fracture. Because the tooth is already fractured, the mechanical behavior of the root after treatment is an important consideration when selecting the obturation materials and technique. Further research is therefore needed to clarify how different root canal filling materials and techniques affect the fracture resistance of roots with incomplete VRFs.

The two control groups were left uninstrumented to evaluate the effect of the simulated incomplete VRF alone on the fracture resistance of intact roots and to provide a reference for the fracture resistance of untreated natural teeth. Each main group was subdivided according to the presence or absence of a simulated incomplete VRF, allowing the effect of the defect to be assessed within each treatment condition.

A standardized model of an incomplete VRF was used in this study. The defect was located on the buccal surface and extended toward the root canal space without involving the lingual wall, because VRFs typically extend buccolingually rather than mesiodistally, most commonly involving the buccal surface [11]. Its length (7.5 mm, representing half the root length) and width (0.25 mm) were consistent with those used by Demiriz and Bodrumlu [10], and its internal and external lengths were equal. The width was deliberately set at 0.25 mm to allow the root canal filling materials to flow into the defect, in line with case reports recommending enlargement and preparation of the fracture line before sealing [13,14]. This approach ensured identical defect dimensions and location across specimens, which is difficult to achieve with naturally propagated cracks. However, a mechanically prepared groove differs from a clinical VRF in several respects: clinical fractures are typically much narrower, have irregular and closely apposed fracture surfaces, and propagate through dentin with a high stress concentration at the crack tip, whereas a cut defect has smooth, parallel walls and involves the removal of dentin. Consequently, stress distribution under load, and possibly the ability of sealers to penetrate and bond within the defect, may differ from those in naturally fractured roots. The present model should therefore be regarded as a simplified simulation, and its results may not be directly extrapolated to clinical VRFs.

The role of the smear layer and its removal with bioceramic sealers remains debated. Some studies propose that the smear layer acts as a coupling agent, enhancing adhesion because bioceramic materials are hydrophilic and set more effectively in the presence of moisture [15]. Conversely, other research suggests that removing the smear layer increases dentin surface energy, facilitating sealer flow into dentinal tubules and enhancing bond strength and root fracture resistance [16]. To standardize dentin surface conditions, the smear layer was removed in this study using 5.25% sodium hypochlorite (NaOCl) and 17% EDTA. EDTA irrigation was limited to one minute to minimize its detrimental effect on dentin structure [17].

Two-way ANOVA revealed no statistically significant differences in fracture resistance among the control (negative and positive) and experimental groups, although the mean values varied. The highest mean value was recorded in untreated roots without a simulated incomplete VRF (A1), which may be explained by the preservation of the entire dentin structure in unprepared roots, as dentin loss during root canal preparation can reduce fracture resistance [18]. However, the mean value of untreated roots with a simulated incomplete VRF (A2) was lower than those of the TotalFill BC Sealer/single-cone groups (B1 and B2). Given the limited sample size and the considerable variability within groups, these numerical differences should not be interpreted as evidence of a difference between groups. Accordingly, neither sealer-technique combination significantly altered root fracture resistance compared with untreated roots, and the present findings do not demonstrate a reinforcing effect of the tested sealers relative to untreated teeth. Nevertheless, the absence of a significant reduction in fracture resistance after canal preparation and obturation may be partly related to the properties of calcium silicate-based sealers, which do not shrink during setting [19] and can form a mineral infiltration zone at the sealer-dentin interface [20], potentially improving adhesion to root dentin [21]. Whether these properties translate into a measurable mechanical benefit requires further investigation with adequately powered designs that include an instrumented-but-unobturated control group.

The lowest mean fracture resistance values were recorded in the TotalFill BC HiFlow Sealer/warm vertical compaction groups (C1 and C2), although the differences were not statistically significant. Because the sealer and the obturation technique were not evaluated independently, the present design cannot determine whether this trend resulted from the obturation technique, the sealer, or their combination. One possible explanation, which remains a hypothesis rather than a finding of this study, relates to warm vertical compaction. The forces exerted by vertical condensers in the apical third, combined with the compaction of thermoplasticized gutta-percha and the heat applied during obturation, may induce stresses and thermal changes in root dentin that could reduce fracture resistance [22]. In addition, the heat generated during this technique may interfere with the setting reaction and bond strength of bioceramic sealers by evaporating moisture within the dentinal tubules [23]. Studies in which sealer type and obturation technique are varied independently are required to test this hypothesis.

No statistically significant effect of the simulated incomplete VRF on fracture resistance was observed, either in the control groups or in the experimental groups. This may be because the simulated defects were incomplete, involving only the buccal surface and extending to half of the root length. Although postoperative radiographs showed radiopaque sealer within the simulated defects, two-dimensional radiographs cannot confirm complete penetration of the sealer, sealing of the defect margins, or bonding between the defect walls. Therefore, the present study cannot determine whether the sealers contributed to the mechanical behavior of the defective roots, and objective evaluation using micro-computed tomography or microscopy is required.

Demiriz and Bodrumlu [10] evaluated the fracture resistance of roots with simulated incomplete VRFs of identical dimensions filled with two calcium silicate-based sealers, MTA Fillapex and Well-Root ST, and reported that both sealers increased fracture resistance, with higher values for Well-Root ST than for MTA Fillapex, possibly owing to the resin content of MTA Fillapex. In contrast, the present study did not demonstrate a significant effect of either sealer-technique combination on fracture resistance. This discrepancy may be related to differences between the two studies in the materials tested, obturation techniques, control groups, loading conditions, and sample size.

This study has several limitations. First, the control roots were neither instrumented nor obturated; therefore, comparisons between the control and experimental groups reflect the combined effects of canal preparation and obturation rather than the effect of the sealer alone. An instrumented-but-unobturated group would have allowed the effect of dentin removal to be distinguished from that of obturation and should be included in future studies. Second, each sealer was applied with a different obturation technique (single-cone for TotalFill BC Sealer and warm vertical compaction for TotalFill BC HiFlow Sealer), so differences between the experimental groups cannot be attributed to the sealer alone. Third, although specimens were standardized by root length, canal anatomy, and minimum dentin thickness and were randomly allocated to groups, root cross-sectional dimensions were not measured or matched among groups, which may have contributed to the variability in fracture load. Fourth, as discussed above, the incomplete VRF was simulated by a mechanically created groove, and the results may differ with other fracture characteristics, including width, length, location, or extent (e.g., a complete fracture involving both root surfaces). Fifth, sealer distribution within the simulated defects was evaluated only on two-dimensional radiographs. Finally, the in vitro design cannot fully reproduce the complexity of the oral environment, including microorganisms, periapical lesions, and dynamic, cyclic masticatory loading, and static loading to failure may not reflect clinical fracture mechanisms. Further studies with larger samples, appropriate instrumented controls, standardized obturation techniques, and cyclic fatigue loading are needed to confirm and extend these findings.

## Conclusions

Within the limitations of this in vitro study, neither TotalFill BC Sealer with single-cone obturation nor TotalFill BC HiFlow Sealer with warm vertical compaction significantly altered root fracture resistance compared with untreated roots, and no statistically significant difference was demonstrated between the two sealer/obturation protocols. The presence of a simulated incomplete VRF had no significant effect on fracture resistance. These findings provide preliminary in vitro data regarding the mechanical behavior of bioceramic-based obturation protocols in roots with simulated incomplete VRFs. Larger studies evaluating sealer and obturation technique independently are needed.
